# Early detection of multiple cancers: the era of methylation-based liquid biopsy

**DOI:** 10.3389/fonc.2026.1850041

**Published:** 2026-06-17

**Authors:** Yajie Lin, Zekai Hu, Leyao Shuai, Zhaowei Tong, Hainv Gao, Junsheng Zhao

**Affiliations:** 1 Bioinformatic Center, Key Laboratory of Artificial Organs and Computational Medicine of Zhejiang Province, Shulan (Hangzhou) Hospital, Shulan International Medical College, Zhejiang Shuren University, Hangzhou, China; 2 Department of Infectious Diseases, Huzhou Central Hospital, Affiliated Central Hospital, Huzhou University, Huzhou, Zhejiang, China; 3 Huzhou Key Laboratory of Precision Medicine Research and Translation for Infectious Diseases, Affiliated Central Hospital, Huzhou University, Huzhou, Zhejiang, China; 4 Department of Infection, Key Laboratory of Artificial Organs and Computational Medicine of Zhejiang Province, Shulan (Hangzhou) Hospital, Shulan International Medical College, Zhejiang Shuren University, Hangzhou, China

**Keywords:** biomarker, circulating tumor DNA (ctDNA), DNA methylation, liquid biopsy, multi-cancer early detection

## Abstract

Liquid biopsy based on circulating cell-free DNA (cfDNA) methylation has become a leading non-invasive strategy for multi-cancer early detection (MCED). Aberrant DNA methylation arises at the early stage of tumorigenesis and displays cancer-type-specific signatures, enabling early capture of tumor-derived epigenetic signals. High-throughput sequencing, digital PCR and machine learning algorithms have greatly improved the sensitivity and specificity of methylation-based assays. Large-scale clinical trials including CCGA, PATHFINDER, THUNDER and GUIDE have validated that MCED tests achieve high specificity (>99%) and reliable accuracy for tissue of origin prediction. Integrating methylomics with fragmentomics further boosts early detection performance, especially for early-stage tumors with low ctDNA shedding. Nevertheless, clinical translation still faces notable hurdles, including technical standardization, biological confounding factors, high cost and the demand for large-scale prospective mortality endpoint validation. Future development will rely on multi-omics integration, optimized bioinformatic pipelines and standardized interventional trials to lower cancer-specific mortality. In summary, methylation liquid biopsy is poised to reshape cancer screening from single-organ late diagnosis to multi-cancer early intervention, offering profound prospects for precision oncology.

## 1 Introduction

Effective and accessible multi-cancer early detection remains a central priority of contemporary oncology research. Aberrant DNA methylation, particularly promoter CpG island hypermethylation, is an early and universal epigenetic hallmark of malignant transformation. Methylation alterations emerge prior to morphological lesion formation and exhibit stable tumor-specific patterns, rendering them ideal candidate biomarkers for non-invasive screening.

Current evidence has validated the conceptual feasibility of methylation-based MCED, yet real-world mortality benefits and standardized clinical workflows remain insufficiently defined. Most reviews focus merely on technical or individual trial outcomes, while lacking systematic comparison of mainstream MCED platforms, in-depth interpretation of biological detection limitations, and actionable clinical guidance for result interpretation and patient management.

This review summarizes the biological basis of cfDNA methylation signatures, mainstream technical platforms, landmark clinical evidence, and inherent translational challenges. We further integrate cross-study comparison, biological confounding mechanisms, and practical clinical decision frameworks, aiming to provide a comprehensive reference for the future implementation and optimization of methylation-driven MCED population screening.

## 2 The emerging paradigm of multi-cancer early detection using methylation signatures

### 2.1 Current landscape of key methylation-based MCED clinical trials

The translation of methylation-based MCED assays from research concepts to clinically applicable tests has required rigorous, large-scale clinical validation studies. These studies have progressively evaluated test performance across diverse populations, cancer types, and clinical contexts, providing increasingly evidence for the potential clinical utility of this approach (**Table 1**).

**Table 1:** Overview of key clinical studies on multi-cancer early detection (MCED) methylation liquid biopsy.

Platform/study	Assay design & sequencing strategy	Multi-omics/additional omics	Bioinformatic feature	Cohort setting (population)	Overall sensitivity	Specificity	Stage I sensitivity	TOO accuracy	Core performance feature & reason for heterogeneity	Ref
Galleri (CCGA Substudy 1)	Targeted bisulfite methylation sequencing	Methylomics only	ML classifier for pan-cancer signature	Western, asymptomatic multi-center	67.30%	99.30%	39.00%	93.00%	Ultra-high specificity; limited Stage I sensitivity due to low ctDNA shedding in early tumors; panel optimized for Western cancer spectrum	Liu et al. (1)
Galleri (CCGA Substudy 3)	Targeted bisulfite methylation sequencing	Methylomics only	Refined ML model	Independent Western cohort	51.50%	99.50%	16.80%	88.70%	Strict independent validation further elevates specificity but lowers overall sensitivity; stage-dependent detection	Klein et al. (2)
PATHFINDER	Galleri targeted methylation assay	Methylomics only	Same CCGA pipeline	US asymptomatic screening population	Not reported	>99%	—	—	Real-world screening setting with low disease prevalence; low PPV (38%) driven by population baseline	Schrag et al. (3)
SYMPLIFY	Methylation-based MCED assay	Methylomics only	ML tissue origin prediction	UK symptomatic referral patients	66.30%	98.40%	24.20%	85.20%	Higher PPV (75.5%) due to higher pre-test disease prevalence in symptomatic cohort	Nicholson et al. (4)
THUNDER	Enhanced linear-splinter amplification methylation sequencing	Methylomics only	Methylation signature modeling	Chinese prospective cohort	69.10%	98.90%	—	83.20%	Optimized for East Asian prevalent cancers (GI, liver, lung); simulation shows strong late-stage reduction benefit	Gao et al. (5)
GutSeer (GUIDE)	Targeted bisulfite sequencing	Methylomics + Fragmentomics	Fragmentomic cleavage profile + methylation integration	Chinese multi-hospital GI cohort	82.80%	95.80%	65.3%–92.9% (GI cancers)	—	Multi-modal integration greatly improves early GI sensitivity; trade-off: slightly lower overall specificity	Huang et al. (6)
INSPECTOR	hTET enzyme-assisted whole-methylome sequencing	Methylomics (enzymatic conversion)	Enzymatic low-degradation library + ML pan-cancer classifier	Multi-cancer validation cohort	81.90%	99.00%	65.50%	—	Enzymatic replacement of bisulfite preserves DNA integrity; superior Stage I sensitivity at maintained high specificity	Luo et al. (7)
SPOGIT	Multi-model cfDNA methylation assay	Methylomics only	Methylation panel for GI cancers	GI cancer multi-center cohort	88.10%	91.20%	83.1% (Stage 0–II)	—	Narrow cancer focus on gastrointestinal tumors yields high early sensitivity but lower universal specificity	Zhu et al. (8)
TOTEM	Targeted ctDNA methylation marker panel	Methylomics only	Minimal diagnostic (21) + CSO (214) marker modeling	Multi-cancer training/test/independent validation cohorts	55.9%–67.3%	98.0%–100%	45.7%–50.3% (I–II)	77.7% (Top1); 86.5% (Top2)	Achieved robust AUC (0.907) with a small number of methylation markers; enables cost-effective multiplex PCR adaptation; stable performance across independent cohorts	Xiong et al. (9)
SPOT-MAS (K-DETEK)	Targeted methylation panel	Methylomics + Fragmentomics	Multi-feature ML integration	Vietnam asymptomatic middle-income population	70.83%	99.71%	73.90%	—	Adapted to non-Western population; ultra-high NPV suitable for mass population screening	Nguyen et al. (10)
Real-world Galleri	Same targeted methylation pipeline	Methylomics only	Deployed clinical ML model	111,080 real-world individuals	—	—	—	87.00%	Real-world performance slightly lower than controlled trial; TOO accuracy remains robust	Matrana et al. (11)

The Circulating Cell-free Genome Atlas (CCGA) study (NCT02889978) represents the foundational clinical validation program for methylation-based MCED testing. This prospective, case-controlled, observational study enrolled participants across multiple centers and substudies to develop and validate a targeted methylation sequencing assay. In the initial substudy, Liu and colleagues evaluated performance across more than 50 cancer types in 6689 participants, demonstrating overall specificity of 99.3% with a false-positive rate of only 0.7%. Stage I-III sensitivity was 67.3% for a pre-specified set of 12 cancer types accounting for approximately 63% of US cancer deaths annually, and sensitivity increased with advancing stage: 39% for stage I, 69% for stage II, 83% for stage III, and 92% for stage IV (1). Importantly, the assay correctly predicted tissue of origin (TOO) in 93% of samples with a detected cancer signal, demonstrating the feasibility of origin localization (1). The subsequent CCGA substudy 3 validated a refined version of this test in an independent cohort of 4077 participants, confirming high specificity of 99.5% and overall sensitivity of 51.5% across all cancer types. The sensitivity for stage I-III cancers in the 12 pre-specified cancer types was 67.6%, and the accuracy of cancer signal origin prediction reached 88.7% (2). Across more than 50 cancer types, cancer signals were detected with increasing sensitivity by stage: 16.8% for stage I, 40.4% for stage II, 77.0% for stage III, and 90.1% for stage IV (2). These results provided the first large-scale validation of a blood-based MCED test for clinical application.

The PATHFINDER study (NCT04241796) represented the first prospective interventional evaluation of MCED testing in a real-world screening context. Conducted across seven US health networks, this study enrolled 6662 participants aged 50 years or older without cancer symptoms. A cancer signal was detected in 1.4% (92/6621) of participants with analyzable results. Among these, 38% (35/92) were subsequently diagnosed with cancer (true positives), yielding a positive predictive value of approximately 38% (3). The median time to diagnostic resolution was 79 days, with shorter times for true-positive participants (57 days) compared to false-positive participants (162 days). Notably, fewer invasive procedures were required for participants with false-positive results (30%) compared to true-positive results (82%), suggesting that diagnostic evaluation following a positive MCED test can be managed without excessive harm (3). This study established the feasibility of integrating MCED testing into clinical screening pathways, while also identifying areas for improvement in diagnostic resolution time and positive predictive value.

The SYMPLIFY study extended MCED evaluation to symptomatic populations, assessing performance in 5461 participants referred for cancer investigation from primary care across England and Wales. In this symptomatic cohort, the MCED test achieved a positive predictive value of 75.5%, negative predictive value of 97.6%, sensitivity of 66.3%, and specificity of 98.4%. Sensitivity increased with age and cancer stage, from 24.2% in stage I to 95.3% in stage IV. TOO prediction accuracy was 85.2% in cases where a cancer signal was detected (4). This study demonstrated the potential of MCED testing to assist clinical decision-making regarding referral urgency and route, particularly for patients presenting with non-specific symptoms that challenge traditional diagnostic pathways.

The THUNDER study (NCT04820868), conducted in China, evaluated an enhanced linear-splinter amplification sequencing approach for detecting six cancer types (colorectum, esophagus, liver, lung, ovary, and pancreas). In a prospective validation cohort of 1010 participants, the model achieved sensitivity of 69.1% with 98.9% specificity and tissue origin accuracy of 83.2%. Importantly, real-world simulation using Chinese cancer incidence data suggested that MCED testing could reduce late-stage incidence by 38.7% to 46.4% and increase five-year survival rates by 33.1% to 40.4% (5). This study provided valuable evidence for the potential population-level impact of MCED screening.

The GUIDE study (NCT05431621, ClinicalTrials.gov identifier: NCT05431621) developed GutSeer, a blood-based assay combining DNA methylation and fragmentomics for multi-gastrointestinal cancer detection. In an independent test cohort of 846 participants across five hospitals, GutSeer achieved an AUC of 0.950 with 82.8% sensitivity and 95.8% specificity for detecting colorectal, esophageal, gastric, liver, and pancreatic cancers. The assay demonstrated high sensitivity for early-stage disease: 92.2% for colorectal, 75.5% for esophageal, 65.3% for gastric, 92.9% for liver, and 88.6% for pancreatic cancers (6). Notably, GutSeer also detected advanced precancerous lesions in the colorectum, esophagus, and stomach, suggesting potential for cancer prevention through identification of premalignant conditions.

Beyond these large-scale MCED studies, numerous cancer-specific methylation assays have demonstrated strong performance for individual cancer types, providing complementary evidence for the approach. For colorectal cancer, the ColonSecure test, based on cfDNA methylation analysis, achieved 86.4% sensitivity at 95.6% specificity in a prospective cohort of 3493 high-risk individuals, substantially outperforming conventional biomarkers including CEA (45.6%), CRP (39.8%), and CA19-9 (25.2%) (12). Similarly, the EpiPanGI Dx assay, targeting gastrointestinal cancers, achieved AUC values of 0.98 for colorectal cancer and 0.98 for hepatocellular carcinoma in cfDNA specimens (13). Another gastrointestinal cancer cohort of 1,079 participants, SPOGIT demonstrated 88.1% sensitivity for GI cancers, 83.1% sensitivity for early-stage (0–II) disease, and 91.2% specificity (8).

The TOTEM approach, which uses methylation markers for multi-cancer detection and localization, achieved an AUC of 0.907 in testing cohorts with 98% specificity, and maintained performance across independent validation cohorts (9).

The K-DETEK study (NCT05227261) provided important evidence from a lower-middle-income country context. Among 9024 asymptomatic individuals aged 40 years or older in Vietnam, the SPOT-MAS MCED test demonstrated positive predictive value of 39.53% and negative predictive value of 99.92%, with overall sensitivity of 70.83% and specificity of 99.71% (14). This study demonstrated that MCED tests could be valuable additions to national screening programs in regions where such initiatives are currently limited.

Real-world evidence from over 100,000 MCED tests has begun to emerge, providing insights into performance outside controlled clinical trial settings. Matrana and colleagues reported that among 111,080 individuals tested with the Galleri MCED test, the cancer signal detection rate was 0.91%. Of 459 individuals with detected cancer signals and reported clinical outcomes, 258 had an invasive cancer diagnosis spanning 32 cancer types. The test correctly predicted the cancer signal origin in 87% of cases, and the median time from result receipt to cancer diagnosis was 39.5 days (11). This large-scale experience provides encouraging evidence for the feasibility and clinical utility of MCED testing in routine practice.

The National Cancer Institute (NCI) established Multi-Cancer Detection (MCD) technology and screening framework for the Vanguard Study (VS) (15). Initial results of this study have recently been reported, showing highly variable detection performance across detection methods and cancer types, and higher sensitivity in advanced cancers than in early cancers (16).

The performance of methylation-based MCED assays is characterized by a consistent pattern: high specificity (typically exceeding 98-99%), stage-dependent sensitivity with lower performance in early-stage disease, and TOO prediction. While overall sensitivity in the range of 50-70% may appear modest, it must be contextualized within the screening paradigm. At 99.5% specificity, a test with 50% sensitivity across more than 50 cancer types would detect substantially more cancers than current screening programs focused on a limited number of organ sites. For cancers with established screening programs, including breast, colorectal, cervical, and lung cancers, MCED tests could serve as a complement rather than a replacement, potentially improving overall detection rates and reducing the burden of late-stage diagnoses. Modeling studies suggest that adding an annual MCED blood test to current screening practices could detect an additional 105,526 to 422,105 cancers annually in the United States, depending on uptake rates (17). The true-positive to false-positive ratio for MCED testing (1:1.8) compares favorably with current screening approaches (1:43), suggesting more efficient diagnostic resource utilization (17). For cancers lacking any screening recommendation, the potential impact of MCED testing is even more profound. *Post-hoc* analysis of the CCGA study demonstrated that aggregate sensitivity for solid tumors without population screening recommendations was 66% across all stages and 53% for stages I-III, with more than 75% sensitivity achieved for 8 of 18 unscreened cancer types (18). Cancers such as pancreatic, ovarian, liver, and head and neck cancers, which are typically diagnosed at advanced stages with dismal prognoses, could potentially be detected at earlier, more treatable stages through MCED screening (18, 19).

The mortality reduction potential of MCED screening has been estimated through modeling studies. Using cancer incidence and survival data from England, one model predicted that screening individuals aged 50–79 years could reduce late-stage cancer diagnoses by 160 to 274 per 100,000 persons, with corresponding mortality reductions of 60 to 99 per 100,000 persons (20). These benefits were greatest in the most socioeconomically deprived groups, suggesting that MCED screening could help reduce cancer health disparities if implemented equitably (20). Another modeling study estimated a 53% reduction in stage IV cancer diagnoses and a gain of 0.38 quality-adjusted life-years among individuals diagnosed with cancer, supporting the potential value-based price of MCED testing at approximately $1,196 per test (21).

### 2.2 Comparative synthesis of major MCED platforms and sources of performance heterogeneity

Performance discrepancies across published methylation-based MCED studies are not merely attributable to cancer stage distribution but are collectively shaped by assay design (targeted vs. enzymatic vs. WGBS), multi-omics integration strategies, bioinformatic feature selection, and cohort population characteristics.

In terms of technical assay design, targeted bisulfite sequencing adopted by the Galleri/CCGA platform achieves exceptional specificity (>99.3%) across more than 50 cancer types via curated cancer-specific CpG panels, yet suffers from relatively low Stage I sensitivity (16.8%–39.0%) due to limited tumor cfDNA shedding in early lesions. In contrast, the INSPECTOR platform replaces traditional bisulfite chemical conversion with hTET enzyme-assisted methylome sequencing, minimizing DNA degradation and retaining intact epigenetic information, thereby elevating Stage I sensitivity to 65.5% while preserving high specificity of 99.0%. Cancer-focused assays such as PDACatch further adopt methylation haplotype analysis, capturing coordinated CpG methylation patterns at single-molecule resolution and outperforming conventional single-CpG averaging for early pancreatic cancer detection.

In terms of multi-omics integration, GutSeer and SPOT-MAS incorporate methylomics combined with fragmentomics, leveraging cfDNA fragmentation profiles as auxiliary biomarkers. This multi-modal strategy markedly improves early-stage detection sensitivity for gastrointestinal and solid tumors, though it moderately reduces overall specificity compared with single-methylomics pan-cancer platforms. By comparison, traditional single-marker methylation assays (THUNDER, SPOGIT) rely solely on methylation signatures, showing stable specificity but limited capability to distinguish weak early-stage tumor signals from inflammatory or age-related epigenetic noise.

Cohort setting and population baseline represent another critical source of heterogeneity. Asymptomatic population screening cohorts (CCGA, PATHFINDER, K-DETEK) have low disease prevalence, leading to stringent specificity thresholds and relatively low positive predictive value (PPV ~38% for PATHFINDER). Symptomatic referral cohorts such as SYMPLIFY enroll patients with clinical suspicion of cancer; higher pre-test prevalence naturally increases PPV to 75.5% without fundamental improvement in analytical performance. Geographical and ethnic differences also drive variation: THUNDER, GUIDE and SPOGIT are optimized for East Asian cancer spectra dominated by gastrointestinal and liver cancers, whereas CCGA and PATHFINDER reflect Western population tumor profiles, resulting in divergent sensitivity in organ-specific cancers.

Collectively, the variability in sensitivity, specificity, early-stage detection rate and TOO accuracy among current MCED platforms is systematically determined by sequencing chemistry, omics modality, bioinformatic feature engineering, and population clinical context rather than stage alone.

## 3 Technical platforms and analytical methodologies

### 3.1 Sample pre-processing and cfDNA baseline characteristics

The technological approaches for measuring cfDNA methylation have evolved substantially (**Supplementary Material**). Bisulfite conversion, which deaminates unmethylated cytosines to uracil while leaving methylated cytosines intact, remains the gold-standard method for methylation analysis. Digital PCR (dPCR) represents a cornerstone of targeted detection, offering exceptional sensitivity for quantifying low-abundance methylated alleles (22). Assay design itself can be optimized; dual-strand dPCR assays that target both DNA strands after bisulfite conversion have been shown to double the detection of methylated DNA copies compared to single-strand assays, thereby improving sensitivity for cancer detection without compromising specificity (23). However, traditional bisulfite sequencing approaches face limitations including DNA degradation and reduced complexity of converted sequences. Modified approaches, including targeted bisulfite sequencing using hybridization capture or amplicon-based methods, have been developed to enrich for methylation markers of interest while reducing sequencing requirements and costs (1, 6).

Enzymatic approaches have emerged as alternatives to bisulfite conversion, offering gentler conditions that preserve DNA integrity. The INSPECTOR study utilized human TET (hTET) enzyme-assisted whole-methylome sequencing, which leverages enzymatic oxidation of 5-methylcytosine rather than chemical conversion, enabling high-depth targeted sequencing with reduced DNA degradation (7). This approach demonstrated strong performance across multiple cancer types, achieving 81.9% sensitivity at 99.0% specificity in an independent validation cohort, with notable sensitivity of 65.5% for stage I cancers (7).

Beyond conventional methylation analysis, additional epigenetic modalities have shown promise. 5-hydroxymethylcytosine (5hmC), an oxidation product of 5-methylcytosine generated by TET enzymes, represents a distinct epigenetic mark with unique biological properties. The Avantect Pancreatic Cancer Test, which leverages 5hmC signatures in cfDNA, has demonstrated strong analytical validity for pancreatic cancer detection, with early-stage sensitivity of 68.3% (24). Zhou and colleagues further showed that 5hmC profiles of long non-coding RNA genes in cfDNA could serve as biomarkers for cancer detection and progression monitoring across multiple cancer types, achieving AUC values exceeding 0.85 in independent validation cohorts (25). TET-assisted pyridine borane sequencing (TAPS) represents another enzymatic approach that specifically detects 5-methylcytosine and 5-hydroxymethylcytosine without bisulfite conversion (26). This method preserves DNA integrity and enables direct detection of modified bases. While TAPS has shown promise in research settings, its application to cfDNA methylation analysis for multi-cancer detection is still in early stages of development.

The methylation haplotype pattern, which examines the coordinated methylation status of multiple CpG sites within individual DNA molecules, has emerged as a particularly informative feature. Unlike single CpG methylation analysis, which averages signals across many molecules, haplotype analysis preserves the epigenetic context of individual cfDNA fragments, potentially providing more reproducible cancer-specific signals. The ColonES assay, which targets 191 genomic regions using methylation haplotype analysis, demonstrated sensitivity of 86.6% for colorectal cancer detection with 88.1% specificity in blinded validation (27).

Understanding the relationship between ctDNA fraction and detection performance has been critical for assay development. Circulating tumor allele fraction (cTAF), which reflects the proportion of tumor-derived cfDNA, is a stronger predictor of classifier performance than either clinical stage or tumor type (26). This finding underscores that assay sensitivity is fundamentally limited by the amount of tumor DNA present in the circulation, which varies substantially across cancer types and stages. Cancers that shed more DNA into the bloodstream, such as liver and colorectal cancers, are more readily detected than those with lower shedding, such as brain and kidney cancers (1, 2).

The translation of DNA methylation biomarkers into clinically viable MCED tests is fundamentally dependent on sensitive technological platforms for analyzing the sparse and fragmented ctDNA signal within a high background of normal cfDNA (28). The landscape of these platforms has evolved significantly, broadly divided into targeted, PCR-based methods and broader, next-generation sequencing-based approaches, each with distinct advantages for sensitivity, multiplexing capability, and cost (29).

While, the NGS-based whole-genome bisulfite sequencing (WGBS) provides an unbiased map of methylation but has been limited by cost and input requirements. To address this, methods like Heatrich-BS selectively enrich and sequence the GC-rich fraction of the genome where cancer-specific methylation signatures are concentrated, enabling scalable tumor burden monitoring and epigenetic subtyping at reduced cost (30, 31). Innovative computational frameworks can also enhance data utility; one method unifies methylation and copy number aberration signals from low-depth WGBS data, using a Bayes model to first enrich ctDNA reads based on hypomethylation haplotypes and then model copy number aberration, achieving substantial signal enrichment for multi-cancer detection (32).

ML is now indispensable for distilling complex, high-dimensional methylation data into clinically actionable predictions. Deep methylation sequencing aided by a ML classifier can detect tumor-derived signals at extreme dilution (0.01%), nearly doubling the detection rate of early-stage lung cancer patients compared to ultradeep mutation sequencing at high specificity (33). A ML model integrating multiple features detected five cancer types with 72.4% sensitivity at 97.0% specificity, demonstrating that multi-feature integration can achieve excellent performance without ultra-deep sequencing (10). In the PROMISE study, a multimodal classifier combining cfDNA methylation and protein markers outperformed a methylation-only classifier, particularly improving the detection cancers (34). The FRAGMA method demonstrated that cfDNA cleavage profiles around CpG sites are highly correlated with methylation status (35). FRAGMAXR was further developed to detect cancer, and its signal is closely related to liver, lung, breast and ovarian cancers (36). Using CG-containing end motifs alone, it achieved high accuracy in differentiating hepatocellular carcinoma patients and improved the positive predictive value in nasopharyngeal carcinoma screening (35). The recent developed METER, a tool for quantification and detection of ctDNA content and molecular subtyping inference using breast cancer-specific DMS and DMRS based on low-pass WGBS of cfDNA (37).

Bisulfite conversion, degrades DNA and obscures sequence complexity. Enzymatic conversion methods offer an alternative that preserves DNA integrity, especially in GC-rich regions, and can be coupled with enrichment strategies like RECAP-seq to improve detection sensitivity for low-abundance ctDNA (38, 39). The use of synthetic spike-in controls for assays like cfMeDIP-seq helps mitigate batch effects and enables absolute quantification (40–42). Large-scale studies have further verified the advantages of combining methylation with fragmentomics, constructing a cfMeDIP-seq dataset including 11 types of cancers and healthy controls, establishing multi-cancer methylation characteristics and cancer-specific signatures (43, 44). Future research directions include the development of more sensitive assays for early-stage detection, integration of additional analyte classes such as proteins and RNA, and validation in diverse populations to ensure equitable performance (45–47). The exploration of alternative biofluids, including urine, cerebrospinal fluid, and saliva, may expand applications to specific cancer types (48, 49). Advances in nanopore sequencing technology offer the potential for direct detection of modified bases without conversion, simplifying workflows and enabling simultaneous analysis of multiple epigenetic marks (50, 51). As the field progresses toward broader implementation, the focus is shifting toward developing cost-effective, highly scalable, yet sensitive platforms, multi-layered information within cfDNA—methylation, fragmentation—to deliver reliable, early, and localized cancer signals.

## 4 Current challenges and considerations for clinical translation

### 4.1 Biological limitations, detection failure and false positive confounders

**Figure 1:**
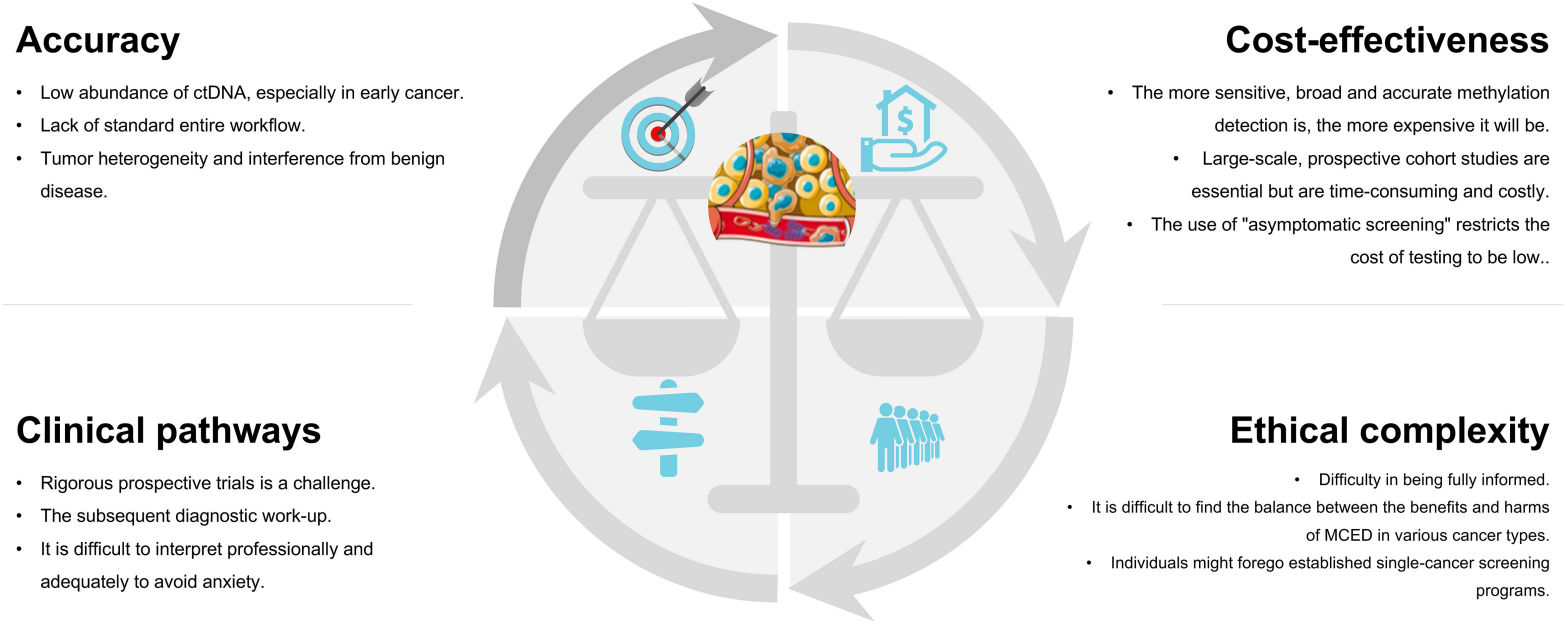
Considerations and balance for clinical translation of methylation-based multi-cancer early detection (MCED).

A fundamental bottleneck limiting the performance of methylation-based MCED lies in the intrinsic biological constraints of tumor-derived cfDNA and widespread non-malignant epigenetic interference (**Figure 1**). Detecting rare tumor-specific methylated fragments amid a high background of normal circulating cfDNA remains technically demanding, particularly for early-stage malignancies with low tumor shedding capacity (31). Across large-scale MCED cohorts, certain cancer types—including brain, renal, and prostate cancers—consistently exhibit persistently low detection sensitivity, which cannot be attributed merely to tumor stage but stems from inherent anatomical and physiological features (1, 2, 52, 53). The intact blood–brain barrier severely restricts the release of brain tumor DNA into peripheral circulation, while renal and prostate tumors typically present low microvessel density, slow cell turnover, and limited apoptotic DNA leakage (54–56). Such biological traits result in an extremely low circulating tumor allele fraction, rendering these malignancies far less amenable to liquid biopsy detection than highly vascularized tumors such as colorectal and liver cancers.

Beyond low ctDNA shedding, biological confounders substantially compromise MCED specificity by generating false-positive methylation signatures that closely resemble cancer-associated epigenetic patterns. Age-related clonal hematopoiesis (CHIP) represents a major source of background noise, in which hematopoietic stem cell clones accumulate aberrant promoter hypermethylation with aging; these altered methylated cfDNA fragments enter the circulation and are frequently misclassified as solid tumor signals (57). Chronic inflammatory conditions, autoimmune disorders, and persistent tissue injury also induce widespread epigenetic reprogramming in normal epithelial and immune cells, triggering aberrant CpG hypermethylation within tumor suppressor genes that overlaps authentic cancer methylation profiles (58). Furthermore, benign adenomas, hyperplastic polyps, and premalignant lesions display intermediate methylation landscapes between normal tissues and invasive cancers, easily triggering false-positive MCED alerts even in the absence of malignant transformation (59). Additionally, tissue-specific baseline methylation variation across individuals creates constitutive background noise, which masks weak early-stage tumor signals and further complicates accurate classification.

To mitigate these biological interferences, multiple complementary strategies have emerged. Establishing age-stratified and population-specific normal methylation baselines enables systematic correction of physiological background noise. Leukocyte and CHIP-specific reference signatures allow computational subtraction of hematopoietic-derived epigenetic noise (60). Integrated multi-omics approaches combining methylomics with fragmentomics, nucleosomal positioning, and protein biomarkers further improve signal discrimination (61). Moreover, methylation haplotype profiling and machine learning-based deconvolution can capture coordinated CpG methylation patterns unique to tumor lineages, reducing misclassification caused by inflammation, aging, and premalignant epigenetic alterations (62).

### 4.2 Technical standardization, analytical bottlenecks and cost-effectiveness

Compounding biological limitations are substantial technical and analytical challenges that hinder consistent clinical translation (63). The entire MCED workflow lacks unified standardization, spanning pre-analytical procedures such as blood collection, plasma processing, cfDNA extraction, and storage conditions, as well as downstream bisulfite conversion, library construction, and sequencing depth configuration (64). Variations in experimental protocols introduce batch effects, distort methylation quantification, and lead to inconsistent performance across institutions and cohorts (65, 66). Traditional bisulfite conversion further compromises data quality by causing partial DNA degradation and reducing sequence complexity, which disproportionately impairs the detection of low-abundance ctDNA fragments in early-stage patients.

Although targeted bisulfite sequencing and WGBS provide powerful methylome profiling, their clinical deployment is constrained by high sequencing costs, excessive data output, and demanding computational resources. While ultra-deep sequencing improves sensitivity, it undermines cost-effectiveness and limits large-scale population screening. By contrast, emerging enrichment strategies, such as Heatrich-BS, cfMeDIP-seq, and RECAP-seq, partially reduce sequencing burden by concentrating cancer-relevant GC-rich methylated regions, yet these methods still lack uniform operational benchmarks and cross-laboratory reproducibility (64, 67). Stable standard analytical pipelines capable of distinguishing subtle tumor-derived signals from the overwhelming presence of nontumor-derived cfDNA are urgently required (68, 69).

DPCR and methylation-specific PCR enable sensitive targeted detection but suffer from limited multiplexing capacity, making them unsuitable for pan-cancer signature profiling. Enzymatic conversion methods, including hTET-assisted sequencing and TAPS, preserve DNA integrity and avoid bisulfite-induced degradation; nevertheless, they face unresolved bottlenecks in reagent scalability, protocol standardization, and widespread clinical compatibility (70–72). Collectively, the absence of unified technical guidelines, coupled with high operational costs and variable analytical reproducibility, remains a central barrier to the widespread implementation of methylation-based MCED as a population screening tool.

### 4.3 Clinical decision framework, post-positive management and integrated screening guidance

Beyond biological and technical hurdles, real-world clinical implementation faces unresolved challenges in standardized post-positive result management, diagnostic pathway design, patient communication, and rational integration with existing screening protocols. Current MCED literature remains largely conceptual, and clinicians lack practical decision-making frameworks for interpreting positive signals, managing cases with unknown tissue origin, and communicating result uncertainty to patients. Evidence from the PATHFINDER and SYMPLIFY trials provides an empirical basis to establish a structured clinical workflow for MCED-positive individuals (3, 4).

For asymptomatic screening participants with a confirmed MCED-positive signal and reliable TOO prediction, a tiered, site-targeted diagnostic workup is recommended, prioritizing non-invasive imaging and serological biomarkers before escalating to endoscopic or pathological verification (73). PATHFINDER demonstrated that true-positive cases achieve diagnostic resolution within a relatively short median interval, whereas false-positive individuals rarely require invasive procedures and can be safely managed through periodic follow-up rather than aggressive intervention (3). For cases with an indeterminate cancer signal of unknown origin, blind extensive imaging and invasive workup should be avoided; instead, a stepwise strategy incorporating age-adapted tumor markers, low-dose whole-body imaging, and serial longitudinal MCED retesting is clinically prudent (74, 75). In symptomatic patients with non-specific constitutional symptoms, as highlighted by the SYMPLIFY study, MCED can effectively stratify disease probability: its high negative predictive value enables safe deferral of unnecessary investigations in MCED-negative individuals, while a positive result accelerates specialist referral and targeted organ-specific evaluation (4).

Clinically, MCED should be clearly differentiated as either an adjunctive population screening tool or a triage-assisted diagnostic tool, rather than a standalone replacement for established screening modalities. In asymptomatic middle-aged and high-risk populations, MCED functions as a complementary annual assay that expands detection coverage for lethal unscreened cancers, including pancreatic, ovarian, liver, and head and neck malignancies, without replacing guideline-recommended mammography, colonoscopy, cervical cytology, or low-dose chest CT (76). In symptomatic patients with unexplained clinical manifestations, MCED serves as a powerful triage tool to streamline ambiguous diagnostic pathways and optimize referral urgency (77).

Equally important is standardized patient counseling regarding result uncertainty. Clinicians should explicitly communicate the stage-dependent sensitivity, ultra-high specificity yet limited positive predictive value, and inherent possibility of false positives or undetermined tissue origin (53). Transparent communication reduces psychological anxiety, avoids overdiagnosis, and helps patients understand that MCED acts as a risk-stratification tool rather than a definitive diagnostic gold standard (53). From a health policy perspective, future integration should prioritize high-risk subgroups defined by age, viral infection status, familial cancer history, and lifestyle exposure, while gradually establishing cost-effectiveness thresholds and standardized diagnostic algorithms to ensure equitable resource allocation and minimize disparities in cancer screening access.

## 5 Conclusions

Methylation-based liquid biopsy for MCED is now at a pivotal transition from exploratory research toward routine clinical application. DNA methylation signatures enable pan-cancer early detection and tissue localization, supported by mature sequencing technologies, artificial intelligence algorithms and accumulating large-scale clinical evidence. Combined multi-omics strategies incorporating fragmentomics continue to break through the detection bottleneck of early-stage low-shedding tumors.

Multiple unresolved challenges still hinder widespread population deployment, including inconsistent technical standardization, biological false-positive interference, cost constraints, and the lack of unified post-positive diagnostic pathways. Additionally, ethical concerns, health equity and reasonable integration with existing cancer screening protocols require deliberate policy and clinical planning.

Future MCED development will focus on low-cost and high-throughput sequencing workflows, enzymatic conversion and nanopore direct detection, as well as multi-omics feature fusion and AI-driven noise deconvolution. Large prospective interventional trials are essential to confirm mortality reduction and define optimal screening populations. With continuous technical iteration and clinical validation, methylation-based MCED will gradually evolve into a standardized, accessible public health tool, facilitating early interceptive therapy and reducing the global burden of late-stage cancers.

## References

[1] Liu MC Oxnard GR Klein EA Swanton C Seiden MV Liu MC . Sensitive and specific multi-cancer detection and localization using methylation signatures in cell-free DNA. Ann Oncol. (2020) 31:745–59. doi: 10.1016/j.annonc.2020.02.011 33506766 PMC8274402

[2] Klein E Richards D Cohn A Tummala M Lapham R Cosgrove D . Clinical validation of a targeted methylation-based multi-cancer early detection test using an independent validation set. Ann Oncol. (2021) 32:1167–77. doi: 10.1016/j.annonc.2021.05.806 34176681

[3] Schrag D Beer T McDonnell C Nadauld L Dilaveri C Reid R . Blood-based tests for multicancer early detection (PATHFINDER): a pros pective cohort study. Lancet. (2023) 402:1251–60. doi: 10.1016/S0140-6736(23)01700-2 37805216 PMC11027492

[4] Nicholson BD Oke J Virdee PS Harris DA O’Doherty C Park JE . Multi-cancer early detection test in symptomatic patients referred for cancer investigation in England and Wales (SYMPLIFY): a large-scale, observational cohort study. Lancet Oncol. (2023) 24:733–43. doi: 10.1016/S1470-2045(23)00277-2 37352875

[5] Gao Q Lin YP Li BS Wang GQ Dong LQ Shen BY . Unintrusive multi-cancer detection by circulating cell-free DNA methylation sequencing (THUNDER): development and independent validation studies☆. Ann Oncol. (2023) 34:486–95. doi: 10.1016/j.annonc.2023.02.010 36849097

[6] Huang A Guo D Su Z Zhong Y Liu L Xiong Z . GUIDE: a prospective cohort study for blood-based early detection of g astrointestinal cancers using targeted DNA methylation and fragmentomi cs sequencing. Mol Cancer. (2025) 24:163. doi: 10.1186/s12943-025-02367-x 40468355 PMC12139136

[7] Luo H Wei W Li P Zhang Q Zhou Z Cui L . The INSPECTOR study: enhanced feasibility for clinical translation of a multi-cancer early detection method based on enzyme-assisted high si gnal-to-noise ratio sequencing of methylated circulating tumor DNA. Cancer Commun Lond. (2025) 45:1645–65. doi: 10.1002/cac2.70071 41165038 PMC12728497

[8] ZhuL LinS XuJ YuJ MenF YuD . Clinical validation of a multi-model blood cfDNA methylation assay for early-stage gastrointestinal cancer screening. J Adv Res. (2025):S209012322500832X. doi: 10.1016/j.jare.2025.10.038 41201494

[9] Xiong D Han T Li Y Hong Y Li S Li X . TOTEM: a multi-cancer detection and localization approach using circulating tumor DNA methylation markers. BMC Cancer. (2024) 24:840. doi: 10.1186/s12885-024-12626-7 39009999 PMC11247868

[10] Nguyen V Nguyen T Doan N Pham T Nguyen G Nguyen T . Multimodal analysis of methylomics and fragmentomics in plasma cell-fr ee DNA for multi-cancer early detection and localization. Elife. (2023) 12:RP89083. doi: 10.7554/eLife.89083 37819044 PMC10567114

[11] Matrana M Shukla V Kingsbury D Poliak M Lipton J McMillin M . Real-world data and clinical experience from over 100,000 multi-cancer early detection tests. Nat Commun. (2025) 16:9625. doi: 10.1038/s41467-025-64094-7 41173830 PMC12578847

[12] Zhao F Bai P Xu J Li Z Muhammad S Li D . Efficacy of cell-free DNA methylation-based blood test for colorectal cancer screening in high-risk population: a prospective cohort study. Mol Cancer. (2023) 22:157. doi: 10.1186/s12943-023-01866-z 37770864 PMC10538018

[13] Kandimalla R Xu J Link A Matsuyama T Yamamura K Parker M . EpiPanGI dx: A cell-free DNA methylation fingerprint for the early detection of gastrointestinal cancers. Clin Cancer Res. (2021) 27:6135–44. doi: 10.1158/1078-0432.CCR-21-1982 34465601 PMC8595812

[14] Nguyen L Nguyen T Le V Bui V Nguyen L Pham N . Prospective validation study: a non-invasive circulating tumor DNA-based assay for simultaneous early detection of multiple cancers in asymptomatic adults. BMC Med. (2025) 23:90. doi: 10.1186/s12916-025-03929-y 39948555 PMC11827191

[15] LeeVan E Skarlupka AL Patriotis C Rubinstein WS Pinsky PF Bolton W . Framework to select multi-cancer detection assays in the national cancer institute’s vanguard study. Cancer Epidemiol Biomarkers Prev. (2025) 34:1787–93. doi: 10.1158/1055-9965.EPI-25-0903 40759003 PMC12491945

[16] Wood ME Pinsky PF Novotny PJ Leevan E Weiss M Edelman DC . Performance of multiple multi-cancer detection tests using a large independent reference set (Alliance A212102). J Natl Cancer Inst. (2026) 118:730–6. doi: 10.1093/jnci/djag001 41499420 PMC13064538

[17] Hackshaw A Cohen SS Reichert H Kansal AR Chung KC Ofman JJ . Estimating the population health impact of a multi-cancer early detection genomic blood test to complement existing screening in the US and UK. Br J Cancer. (2021) 125:1432–42. doi: 10.1038/s41416-021-01498-4 34426664 PMC8575970

[18] Shao SH Allen B Clement J Chung G Gao J Hubbell E . Multi-cancer early detection test sensitivity for cancers with and without current population-level screening options. Tumori. (2023) 109:335–41. doi: 10.1177/03008916221133136 36316952 PMC10248281

[19] Vittone J Gill D Goldsmith A Klein EA Karlitz JJ . A multi-cancer early detection blood test using machine learning detects early-stage cancers lacking USPSTF-recommended screening. NPJ Precis Oncol. (2024) 8:91. doi: 10.1038/s41698-024-00568-z 38632333 PMC11024170

[20] Smittenaar R Quaife S von Wagner C Higgins T Hubbell E Lee L . Impact of screening participation on modelled mortality benefits of a multi-cancer early detection test by socioeconomic group in England. J Epidemiol Community Health. (2024) 78:345–53. doi: 10.1136/jech-2023-220834 38429085 PMC11103338

[21] Tafazzoli A Ramsey S Shaul A Chavan A Ye W Kansal A . The potential value-based price of a multi-cancer early detection genomic blood test to complement current single cancer screening in the USA. Pharmacoeconomics. (2022) 40:1107–17. doi: 10.1007/s40273-022-01181-3 36038710 PMC9550746

[22] Zhao Y O’Keefe C Hu J Allan C Cui W Lei H . Multiplex digital profiling of DNA methylation heterogeneity for sensitive and cost-effective cancer detection in low-volume liquid biopsies. Sci Adv. (2024) 10:eadp1704. doi: 10.1126/sciadv.adp1704 39576863 PMC11584010

[23] Jensen S Øgaard N Nielsen H Bramsen J Andersen C . Enhanced performance of DNA methylation markers by simultaneous measurement of sense and antisense DNA strands after cytosine conversion. Clin Chem. (2020) 66:925–33. doi: 10.1093/clinchem/hvaa100 32460325

[24] Chowdhury S Kesling M Collins M Lopez V Xue Y Oliveira G . Analytical validation of an early detection pancreatic cancer test using 5-hydroxymethylation signatures. J Mol Diagn. (2024) 26:888–96. doi: 10.1016/j.jmoldx.2024.06.007 39230538

[25] Zhou M Hou P Yan C Chen L Li K Wang Y . Cell-free DNA 5-hydroxymethylcytosine profiles of long non-coding RNA genes enable early detection and progression monitoring of human cancers. Clin Epigenet. (2021) 13:197. doi: 10.1186/s13148-021-01183-6 34689838 PMC8543867

[26] Jamshidi A Liu MC Klein EA Venn O Hubbell E Beausang JF . Evaluation of cell-free DNA approaches for multi-cancer early detection. Cancer Cell. (2022) 40:1537–1549.e12. doi: 10.1016/j.ccell.2022.10.022 36400018

[27] Mo S Dai W Wang H Lan X Ma C Su Z . Early detection and prognosis prediction for colorectal cancer by circulating tumour DNA methylation haplotypes: A multicentre cohort study. EClinicalMedicine. (2023) 55:101717. doi: 10.1016/j.eclinm.2022.101717 36386039 PMC9646872

[28] Wu H Wang M Wan Z Yao L Zhou S Wang H . Development and validation of a high-confidence diagnostic model integrating ctDNA methylation and serum biomarkers for early-stage hepatocellular carcinoma detection. Mol BioMed. (2026) 7:26. doi: 10.1186/s43556-026-00426-3 41807813 PMC12976210

[29] Luo H Wei W Ye Z Zheng J Xu R . Liquid biopsy of methylation biomarkers in cell-free DNA. Trends Mol Med. (2021) 27:482–500. doi: 10.1016/j.molmed.2020.12.011 33500194

[30] Cheruba E Viswanathan R Wong P Womersley H Han S Tay B . Heat selection enables highly scalable methylome profiling in cell-free DNA for noninvasive monitoring of cancer patients. Sci Adv. (2022) 8:eabn4030. doi: 10.1126/sciadv.abn4030 36083902 PMC9462700

[31] Zhao J Shen S Zhang J Xu Y Peng J Gao H . Whole-genome bisulfite sequencing identifies blood-based DNA methylation biomarker for hepatocellular carcinoma. Mol Carcinog. (2026) 65:603–14. doi: 10.1002/mc.70101 41764773 PMC13067798

[32] Ning W Wu T Wu C Wang S Tao Z Wang G . Accurate prediction of pan-cancer types using machine learning with minimal number of DNA methylation sites. J Mol Cell Biol. (2023) 15:mjad023. doi: 10.1093/jmcb/mjad023 37037781 PMC10635511

[33] Liang N Li B Jia Z Wang C Wu P Zheng T . Ultrasensitive detection of circulating tumour DNA via deep methylation sequencing aided by machine learning. Nat BioMed Eng. (2021) 5:586–99. doi: 10.1038/s41551-021-00746-5 34131323

[34] Duan J Gao Q Wang Z Xu J Zhang Y Wang Y . Exploration of multi-omics liquid biopsy approaches for multi-cancer early detection: The PROMISE study. Innovation Camb. (2026) 7:101076. doi: 10.1016/j.xinn.2025.101076 41737326 PMC12925926

[35] Zhou Q Kang G Jiang P Qiao R Lam WKJ Yu SCY . Epigenetic analysis of cell-free DNA by fragmentomic profiling. Proc Natl Acad Sci. (2022) 119:e2209852119. doi: 10.1073/pnas.2209852119 36288287 PMC9636966

[36] Zhu G Jiang P Li X Peng W Choy LYL Yu SCY . Methylation-associated nucleosomal patterns of cell-free DNA in cancer patients and pregnant women. Clin Chem. (2024) 70:1355–65. doi: 10.1093/clinchem/hvae118 39206580

[37] Paoli M Galardi F Nardone A Biagioni C Romagnoli D Di Donato S . A computational framework for sensitive tumor detection and accurate subtyping using shallow cell-free DNA methylome sequencing. Genome Med. (2026) 18:27. doi: 10.1186/s13073-026-01603-3 41634848 PMC12958694

[38] Shin D Kim T Lee J Kim H Kim T Bang D . RECAP-seq: restriction enzyme-based CpG-methylated fragment amplification for early cancer detection. Sci Rep. (2025) 15:40892. doi: 10.1038/s41598-025-24708-y 41258415 PMC12630582

[39] Shen J Ren Y Mao Z Hu Q Wan Y Du J . Improved circulating tumor DNA identification for detection of esophageal squamous cell carcinoma by enzymatic methyl sequencing and hybrid neural network. Sci Rep. (2025) 15:33004. doi: 10.1038/s41598-025-18278-2 41006474 PMC12475058

[40] Hua X Zhou H Wu H-C Furnari J Kotidis CP Rabadan R . Tumor detection by analysis of both symmetric- and hemi-methylation of plasma cell-free DNA. Nat Commun. (2024) 15:6113. doi: 10.1038/s41467-024-50471-1 39030196 PMC11271492

[41] Qi J Hong B Tao R Sun R Zhang H Zhang X . Prediction model for Malignant pulmonary nodules based on cfMeDIP‐seq and machine learning. Cancer Sci. (2021) 112:3918–23. doi: 10.1111/cas.15052 34251068 PMC8409309

[42] Wilson S Shen S Harmon L Burgener J Triche TJ Bratman S . Sensitive and reproducible cell-free methylome quantification with synthetic spike-in controls. Cell Rep Methods. (2022) 2:100294. doi: 10.1016/j.crmeth.2022.100294 36160046 PMC9499995

[43] Nassiri F Chakravarthy A Feng S Shen SY Nejad R Zuccato JA . Detection and discrimination of intracranial tumors using plasma cell-free DNA methylomes. Nat Med. (2020) 26:1044–57. doi: 10.1038/s41591-020-0932-2 32572265 PMC8500275

[44] Zeng Y Abelman DD Singhawansa A Cheng N Fang Y Main SC . A pan-cancer compendium of 1,294 plasma cell-free DNA methylomes and fragmentomes enabling multicancer detection. Nat Cancer. (2026) 7:384–98. doi: 10.1038/s43018-026-01116-3 41714824 PMC12948677

[45] Zhang K Fu R Liu R Su Z . Circulating cell-free DNA-based multi-cancer early detection. Trends Cancer. (2024) 10:161–74. doi: 10.1016/j.trecan.2023.08.010 37709615

[46] Anees M Sherry C Park H Grayhack E Goel A Khan A . Liquid biopsy biomarkers for early detection of gastrointestinal cancers: Current landscape and emerging technologies. Clin Transl Med. (2026) 16:e70594. doi: 10.1002/ctm2.70594 41866828 PMC13093818

[47] Zhu H Li Z Xie K Kassim S Cao C Huang K . Liquid biopsy in early screening of cancers: emerging technologies and new prospects. Biomedicines. (2026) 14:158. doi: 10.3390/biomedicines14010158 41595692 PMC12839035

[48] Nikanjam M Kato S Kurzrock R . Liquid biopsy: current technology and clinical applications. J Hematol Oncol. (2022) 15:131. doi: 10.1186/s13045-022-01351-y 36096847 PMC9465933

[49] Kim T Shin D Ahn H Moon Y Bang D Kim K . Urine-based cfDNA ensemble modeling for early detection of bladder cancer using whole-genome methylation sequencing. Cancers Bsl. (2026) 18:767. doi: 10.3390/cancers18050767 41827701 PMC12984421

[50] Sanchez-Delgado M Frank M Šišmiš T Kahraman M Daniel-Moreno A Mummery E . Nanopore based RNA methylation profiling of a circulating lung cancer biomarker. Commun Med Lond. (2025) 5:521. doi: 10.1038/s43856-025-01235-5 41402562 PMC12708728

[51] You J Shen S Fan J Zhen S Sha J . Clinical translation of ctDNA epigenetic signatures in lung cancer: An integrated strategy for early detection, therapeutic guidance, and prognostic stratification. Biochem Biophys Res Commun. (2025) 783:152636. doi: 10.1016/j.bbrc.2025.152636 40946553

[52] Mahal B Margolis M Hubbell E Chen C Venstrom J Abran J . A targeted methylation-based multicancer early detection blood test pr eferentially detects high-grade prostate cancer while minimizing overd iagnosis of indolent disease. JCO Precis Oncol. (2024) 8:e2400269. doi: 10.1200/PO.24.00269 39208374 PMC11371104

[53] Rendek T Pos O Duranova T Saade R Budis J Repiska V . Current challenges of methylation-based liquid biopsies in cancer diag nostics. Cancers Bsl. (2024) 16:2001. doi: 10.3390/cancers16112001 38893121 PMC11171112

[54] Friedman JS Hertz CAJ Karajannis MA Miller AM . Tapping into the genome: the role of CSF ctDNA liquid biopsy in glioma. Neuro-Oncol Adv. (2022) 4:ii33–40. doi: 10.1093/noajnl/vdac034 36380863 PMC9650472

[55] Tretiakova M Antic T Binder D Kocherginsky M Liao C Taxy JB . Microvessel density is not increased in prostate cancer: digital imaging of routine sections and tissue microarrays. Hum Pathol. (2013) 44:495–502. doi: 10.1016/j.humpath.2012.06.009 23069258

[56] Iakovlev VV Gabril M Dubinski W Scorilas A Youssef YM Faragalla H . Microvascular density as an independent predictor of clinical outcome in renal cell carcinoma: an automated image analysis study. Lab Invest. (2012) 92:46–56. doi: 10.1038/labinvest.2011.153 22042086

[57] Abbosh C Swanton C Birkbak NJ . Clonal haematopoiesis: a source of biological noise in cell-free DNA analyses. Ann Oncol. (2019) 30:358–9. doi: 10.1093/annonc/mdy552 30649226 PMC6442654

[58] Surace AEA Hedrich CM . The role of epigenetics in autoimmune/inflammatory disease. Front Immunol. (2019) 10. doi: 10.3389/fimmu.2019.01525 31333659 PMC6620790

[59] Wynter CVA Walsh MD Higuchi T Leggett BA Young J Jass JR . Methylation patterns define two types of hyperplastic polyp associated with colorectal cancer. Gut. (2004) 53:573–80. doi: 10.1136/gut.2003.030841 15016754 PMC1774017

[60] Seale K Teschendorff A Reiner AP Voisin S Eynon N . A comprehensive map of the aging blood methylome in humans. Genome Biol. (2024) 25:240. doi: 10.1186/s13059-024-03381-w 39242518 PMC11378482

[61] Dugué P-A Bodelon C Chung FF Brewer HR Ambatipudi S Sampson JN . Methylation-based markers of aging and lifestyle-related factors and risk of breast cancer: a pooled analysis of four prospective studies. Breast Cancer Res. (2022) 24:59. doi: 10.1186/s13058-022-01554-8 36068634 PMC9446544

[62] Liang W-W Lu R-H Jayasinghe RG Foltz SM Porta-Pardo E Geffen Y . Integrative multi-omic cancer profiling reveals DNA methylation patterns associated with therapeutic vulnerability and cell-of-origin. Cancer Cell. (2023) 41:1567–1585.e7. doi: 10.1016/j.ccell.2023.07.013 37582362 PMC11613269

[63] Shen H Jin Y Zhao H Wu M Zhang K Wei Z . Potential clinical utility of liquid biopsy in early-stage non-small c ell lung cancer. BMC Med. (2022) 20:480. doi: 10.1186/s12916-022-02681-x 36514063 PMC9749360

[64] Shin S Woo HI Kim J-W Kim Y Lee K-A . Clinical practice guidelines for pre-analytical procedures of plasma epidermal growth factor receptor variant testing. Ann Lab Med. (2022) 42:141–9. doi: 10.3343/alm.2022.42.2.141 34635607 PMC8548242

[65] Gall T Belete S Khanderia E Frampton A Jiao L . Circulating tumor cells and cell-free DNA in pancreatic ductal adenoca rcinoma. Am J Pathol. (2019) 189:71–81. doi: 10.1016/j.ajpath.2018.03.020 30558725

[66] Geertsen L Koldby K Thomassen M Kruse T Lund L . Circulating tumor DNA in patients with renal cell carcinoma. A systema tic review of the literature. Eur Urol Open Sci. (2022) 37:27–35. doi: 10.1016/j.euros.2021.12.006 35106503 PMC8784339

[67] de Abreu AR Ibrahim J Lemonidis V Mateiu L Van Camp G Op de Beeck K . Comparison of current methods for genome-wide DNA methylation profiling. Epigenet Chromatin. (2025) 18:57. doi: 10.1186/s13072-025-00616-3 40855329 PMC12376410

[68] Jeong S Go D Jeon Y Kim Y-J Lee H Kim Y-W . Enhanced multicancer screening assay through whole-genome methylation sequencing-based multimodal cell-free DNA analysis. Exp Mol Med. (2026) 58:1311–24. doi: 10.1038/s12276-026-01674-7 42014847 PMC13144671

[69] Kim SY Jeong S Lee W Jeon Y Kim Y-J Park S . Cancer signature ensemble integrating cfDNA methylation, copy number, and fragmentation facilitates multi-cancer early detection. Exp Mol Med. (2023) 55:2445–60. doi: 10.1038/s12276-023-01119-5 37907748 PMC10689759

[70] Nuttall B Karl DL Burke K Callahan M Mendler K Cingolani P . Comprehensive comparison of enzymatic and bisulfite DNA methylation analysis in clinically relevant samples. Clin Epigenet. (2025) 17:156. doi: 10.1186/s13148-025-01959-0 41044668 PMC12495756

[71] Simons RB Karkala F Kukk MM Adams HHH Kayser M Vidaki A . Comparative performance evaluation of bisulfite- and enzyme-based DNA conversion methods. Clin Epigenet. (2025) 17:56. doi: 10.1186/s13148-025-01855-7 40181442 PMC11969950

[72] Kresse SH Thorkildsen EM Brandt-Winge S Pharo H Vedeld HM Lind GE . Comparison of enzymatic and bisulfite conversion of circulating cell-free tumor DNA for DNA methylation analyses. Clin Epigenet. (2025) 17:93. doi: 10.1186/s13148-025-01901-4 40468374 PMC12135323

[73] Dang Nguyen LH Tieu BL Nguyen TT Ha NP Huong Nguyen GT Hanh Nguyen TH . A consultation and work-up diagnosis protocol for a multicancer early detection test: a case series study. Future Sci OA. (2024) 10:2395244. doi: 10.1080/20565623.2024.2395244 39254097 PMC11389743

[74] Massart M Raoof S Hubbell E Klein EA . Molecular cancer signal localization in MCED testing minimizes radiation and imaging burden compared to whole body imaging approaches. Cancer Prev Res Phila. (2026) 19:353–60. doi: 10.1158/1940-6207.CAPR-25-0283 41996412 PMC13223538

[75] Marinac CR McDonnell CH Nadauld LD Dilaveri CA Reid R Chung KC . Clinical evaluation of cancer signal origin prediction and diagnostic resolution following multicancer early detection testing in the PATHFINDER study. Cancer Prev Res Phila. (2025) 18:475–83. doi: 10.1158/1940-6207.CAPR-24-0468 40265568 PMC12314506

[76] Sempere LF . Ethical considerations and implications of multi-cancer early detectio n screening: Reliability, access and cost to test and treat. Cambridge Q Healthcare Ethics. (2025) 34:489–98. doi: 10.1017/S0963180124000744 39749955 PMC12703720

[77] Callender T Mackie A Slowther A-M . The ethics of multi-cancer screening. Nat Med. (2026) 32:407–9. doi: 10.1038/s41591-025-04111-w 41491105

